# *Tle4z1* Facilitate the Male Sexual Differentiation of Chicken Embryos

**DOI:** 10.3389/fphys.2022.856980

**Published:** 2022-04-07

**Authors:** Chen Chen, Shujian Zhou, Ziyi Lian, Jingyi Jiang, Xiaomin Gao, Cai Hu, Qisheng Zuo, Yani Zhang, Guohong Chen, Kai Jin, Bichun Li

**Affiliations:** ^1^Key Laboratory of Animal Breeding Reproduction and Molecular Design for Jiangsu Province, College of Animal Science and Technology, Yangzhou University, Yangzhou, China; ^2^Joint International Research Laboratory of Agriculture and Agri-Product Safety of Ministry of Education of China, Yangzhou University, Yangzhou, China; ^3^College of Biotechnology, Jiangsu University of Science and Technology, Zhenjiang, China

**Keywords:** chicken, sex differentiation, *Tle4z1*, male, gene

## Abstract

**Background:**

Sex differentiation is a complex and precisely regulated process by multiple genes in chicken. However, it is still unclear on the key genes of sex differentiation.

**Objective:**

To explore the function of *Tle4z1* screened by RNA-seq sequencing on sex differentiation during the development of chicken embryos.

**Methods:**

*Tle4z1* was differentially expressed from the RNA-seq of ESCs and PGCs in male and female chickens. Then, we established an effective method to overexpression or knocking down the expression of *Tle4z1* in ovo and *in vitro*, respectively. Histomorphological observation, qRT-PCR and ELISA were applied to detect the function of *Tle4z1* in the process of male sex differentiation by injecting vectors into embryos at day 0.

**Results:**

It showed that *Tle4z1* has significant male preference in embryonic day 4.5, such phenomenon persisted during the growth period of chicken embryos. Morphological observation results showed that the gonads on both sides of genetic male (ZZ) embryos with *Tle4z1* knocking down developed asymmetrically, the gonadal cortex became thicker showing the typical characteristics of genetic female (ZW) gonads. Furthermore, the expression of Cyp19a1, which dominates female differentiation, was significantly increased, while the expression of male marker genes Dmrt1, Sox9, WT1 and AR was significantly downregulated. In addition, the concentration of testosterone also significantly decreased, which was positively correlated with the expression of *Tle4z1* (*P* < 0.01). Conversely, the ZW embryo showed defeminized development when *Tle4z1* was overexpressed.

**Conclusion:**

We prove that the *Tle4z1* is a novel gene through the male sexual differentiation *via* gene regulation process and synthesis of testosterone, which construct the basis for understanding the molecular mechanism of sex differentiation in chickens.

## Introduction

Gender control technology is an important means to affect the economic development of livestock and poultry industry. The development of this technology can achieve single-sex directional breeding in the early sex differentiation of poultry to produce meat and eggs products with different gender needs. Existing studies have shown that the mode of sex determination is genotypic sex determination (GSD) in chicken (Wilhelm et al., 2007), but the molecular mechanism of sex differentiation is not clear, which has become an important factor restricting monosex culture of chicken. Therefore, exploring the key genes in the process of sex differentiation and investigating its biological functions are the basic premise to clarify the molecular mechanism of sex differentiation. Studies show that *Dmrt1* located on Z chromosome can regulate gonadal differentiation in chicken. When knockdown *Dmrt1* by RNA interference, it showed that the expression of DMRT1 decreased *in vivo* (Smith and Sinclair, 2004), hereditary male (ZZ) embryos showed feminized development, while the expression of *Sox9* was downregulated, which confirmed that DMRT1 can regulate the expression of *Sox9*, and both jointly regulate testicular development (Hirst et al., 2018; Yamashita et al., 2019). Anti-Müllerian hormone was found to be expressed in both sexes of chicken embryos before Sox9. And the upregulation of sox9 expression will increase the secretion of anti-mullerian hormone expression (Oreal et al., 1998; Lan et al., 2013). Moreover, WT1 was found to promote the formation of sertoli cells, but all above genes were not the sex differentiation switching factors in chicken (Wen et al., 2016). With the in-depth study of sex determination and sex differentiation in poultry, the theories of cell-autonomous, hormone effect on sex determination, hypothesis of the dose effect of Z chromosome and the dominant effect of W chromosome have been put forward (Scheib, 1983; Lin et al., 1995; Graves, 2014; Zimmer et al., 2016). The essence of these hypotheses is to control the process of sex development by regulating the expression of downstream genes, so it is very important to screen the key genes in the process of sex determination and differentiation.

Based on the existing research, the research used ESCs and PGCs as materials for RNA-seq. To obtain the differentially expressed genes between the sexes during the ESCs and PGCs periods, the data is analyzed for grouping and comparison by Venn. According to its chromosome location, the key candidate gene *Tle4z1*, for sex differentiation on chicken Z chromosome was screened and its function in the process of sex differentiation was explored. *Tle4z1* (transducin like enhancer of split4), a member of Tle/gro family, which acts as a co-factor, is specifically and highly expressed in embryonic stem cells and play an important role in early embryonic differentiation (Chen and Courey, 2000). Tle4 in mouse was discovered to promote embryonic stem cell differentiation (Laing et al., 2015). Gro is a transcriptional corepressor recruited to specific target promoters by hairy-related bHLH proteins to affect neurogenesis, segmentation and sex determination in Drosophila *in vivo* maternal requirements (Paroush et al., 1994). Meanwhile, Tle4, a member of the Tle/Gro family, has an effect on the asymmetric development of dorsal ventral patterns in vertebrates (Buscarlet and Stifani, 2007). However, the role of *Tle4z1* on chicken Z chromosome in the process of sex determination and sex differentiation has not been reported.

The basis of the results in transcriptome sequencing, the key candidate gene *Tle4z1* for male differentiation of chicken embryo was screened. To analyze the function of *Tle4z1* in the process of chicken embryo sex differentiation, RNA interference and overexpression system was used to explore the direction of gonadal phenotypic differentiation, sex-related genes and testosterone expression changes after interference (ZZ embryo) and overexpression (ZW embryo) of *Tle4z1* in chicken.

## Materials and Methods

### Ethics Statement and Experimental Animals

The procedures involving animals and their care conformed to the United States National Institute of Health guidelines (NIH Pub. No. 85–23, revised 1996). The experiments were conducted under the approval of the Ethics Committee of Yangzhou University for Laboratory and Experimental Animals. The fertilized eggs of Rugao yellow chicken were purchased from the Poultry Research Institute of Chinese Academy of Agricultural Sciences and hatched under the environment of 37°C with 70% humidity.

### Sample Collection

The experimental eggs were hatched after 0 days of blunt-end injection. A total of three groups were divided into blank, sh-*Tle4z1* and oe-*Tle4z1* group, with 140 fertilized eggs in each group. Embryos at different stages were collected according to the chicken embryo development map (Hamburger Hamilton stage) and stored in −80°C for gonadal isolation and extraction.

The organs, such as heart, liver, spleen, lung, kidney, intestine, eye, ear, brain and gonad of chickens were collected from healthy roosters and hens, which growing in great feeding conditions, and were used to detect the expression profile of *Tle4z1* organs. Blood samples were taken for sex identification.

### Isolation of Chicken ESCs and PGCs

ESCs were derived from E0 chicken embryos and PGCs from the genital ridge of embryos at E4.5. All eggs were incubated under the environment of 37.5°C and 65% relative humidity. The separation and cultivation detailed steps of ESCs and PGCs are introduced in previous article (Zhang et al., 2015). Isolated ESCs and PGCs were used to detect the expression of *Tle4z1* by qRT-PCR.

### Construction of sh-*Tle4z1* Lentivirus Interference Vector

According to the chicken *Tle4z1* (NM_204237.1) gene sequence provided by Genebank, the RNA interference target sequence was designed (Supplementary Table 1), and the oligo sequence was synthesized(Supplementary Table 2). After annealing, the double-stranded DNA vector was formed. The ligated products were transformed into Escherichia coli supercells overnight culture, positive single bacteria were sequenced, and the correct sequences were compared. The plasmid was extracted and packaged with lentivirus (5 × 10^8^TU/ml). The vector was transfected into DF-1 cells and the fluorescence expression was observed 72 h. The RNA was reverse transcribed into cDNA, and the interference efficiency was detected by qRT-PCR.

### Construction of oe-*Tle4z1* Lentivirus Overexpression Vector

The full-length cDNA sequence of *Tle4z1* was obtained by using the cDNA of chicken PGCs as template. The amplification primer sequence is as follows: F: ATGATCCGCGACCTGAGCAAG; R: CTAATAAATAACTTCATAGACTGTAGCTTTCTTGTCCC CA. The target fragment was ligated with the enzyme-digested pCDH-CMV-MCS-EF1-TagRFP + Puro, and the competent cells were transformed and cultured overnight. The positive colonies were sequenced, and the plasmids were extracted for lentivirus packaging (1 × 10^8^TU/ml). The vector was transfected into DF-1 cells, and the overexpression efficiency was detected.

### Blunt Injection of Chicken Embryo

The MOI of lentiviral vector was optimized to be 10. The lentivirus concentrate of overexpression group (oe-*Tle4z1*) and interference group (sh-*Tle4z1*) was diluted with DMEM according to the ratio of 100 μL of each egg to the blunt end of E0. After being sealed with paraffin, the eggs were incubated normally at 38°C.

### Sex Identification

Genomic DNA from blood was extracted to identify the sex of the embryo. Amplification was performed using CHD1 specific primers (CHD1-F: CTGCGAGAACGTGGCAACAGAGT; CHD1-R: ATTGAAATGATCCAGTGCTTG). The results showed that the hereditary female (ZW) had two strips at 580 and 423 bp, while the hereditary male (ZZ) had only one strip at 580 bp.

### Paraffin Sections and Hematoxylin-Eosin Staining

The embryonic gonad of E18.5 was fixed with 4% paraformaldehyde for 24 h, and transferred to 70–100% ethanol was dehydrated. The tissue was embedded in paraffin after being immersed in xylene for 1 h. The paraffin tissue was continuously sliced with a thickness of 5–6 μm and baked at 55–60°C overnight. The slices were dewaxed with xylene and the ethanol concentration was permeated with water from high to low gradient. The treated slices were stained, soaked in hematoxylin dye for 2.5 min, rinsed under flowing water for 10 min, then soaked in eosin staining for 3–5 min, rinsed with flowing water for 5 min, dehydrated and sealed. The sections were observed under microscope.

### Morphological Statistics

The intact fetal gonads were taken. The hereditary female and male gonads of the control group, 15 pairs of male gonads of the interference group and female gonads of the overexpression group were set. The morphology was taken under stereoscopic microscope, and the surface area of gonads was measured, and the ratio of surface area of bilateral gonads was calculated.

### qRT-PCR

Total RNA was extracted from tissues and cells to synthesize cDNA by reverse transcription. The internal reference gene: β-actin, *n* = 3. And primers are shown in the Supplementary Table 3. The PCR reaction was carried out in the condition of 95°C 30 s, 95°C 10 s, 60°C 30 s, 40 cycles.

### ELISA

The gonads at different developmental stages were weighed and crushed with PBS, and the supernatant was taken after 3,000 rpm centrifugation for 25–30 min at 4°C. The hormone of testosterone was detected with an ELISA kit for color development, and the absorbance value was detected at 450 nm. According to the concentration and OD value, the testosterone content of each group was analyzed.

### Statistics

Relative gene expression was calculated using the 2-ΔΔCt method after qPCR. All experiments were performed in triplicate, and the data are presented as mean ± standard error. Significant differences between the groups were determined with two-sample *t*-tests in SPSS 17.0. (*P < 0.05, significant difference. ^**^P < 0.01, extremely significant difference, ^***^P < 0.001, ^****^P < 0.0001). Prism7 software was used for mapping.

## Results

### Screening of *Tle4z1*, a Key Candidate Gene for Sex Differentiation

**FIGURE 1:**
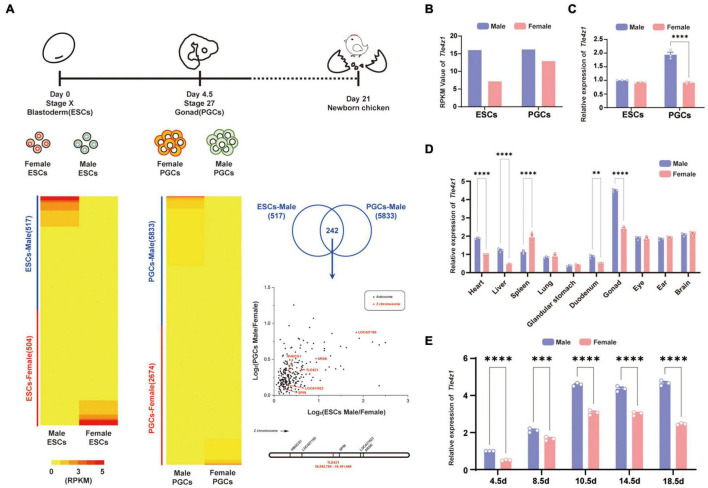
The sexually dimorphic expression of *Tle4z1* in cells and tissues of chicken. **(A)** Experimental design of RNA-seq analysis (top). RPKM of differentially expressed genes(DEGs) in ESCs and PGCs of different genders (left bottom). Filter out male-specific high expression genes of Z chromosome (right). Venn analysis screened out 242 DEGs shared in male PGCs and ESCs. Volcano plot where the x-axis represents the level of differential expression of PGCs and the y-axis shows significant differences in expression of ESCs in both sexes. Genes in Z chromosome are indicated by red dots, and autosome chromosome genes are indicated by black dots. The diagram marks the locations of six male highly-expressed genes (right bottom). **(B)** RPKM of *Tle4z1* in ESCs and PGCs of different genders. **(C)** Validation of mRNA expression of *Tle4z1* by qRT-PCR. The expression of *Tle4z1* in male ESCs was defined as 1 after normalization to β-actin. **(D)** Relative expression of *Tle4z1* in adult chicken tissues as measured by qRT-PCR. **(E)** Relative expression of *Tle4z1* during the development of chicken embryos by extraction of RNA from gonads at different stages (E4.5-E18.5). ^**^P < 0.01, ^***^P < 0.001, ^****^P < 0.0001. All values are mean ± SEM. *n* = 3.

At different development stages of chicken embryos, germ cells were carried out by RNA-seq (Jin et al., 2020a). According to the result, 1021 differentially expressed genes (DEGs) were found in ESCs, including 517 DEGs in males and 504 DEGs in females, while 8,057 differentially expressed genes in PGCs, including 5,833 DEGs in males and 2,674 DEGs in females. In order to further study, the data of transcriptome sequencing between ESCs and PGCs between male and female were analyzed by Venn to find key candidate genes in the process of male sexual differentiation in chicken. 242 DEGs were screened. Map these genes on the chromosomes and watch out that there are six male highly expressed genes located on the Z chromosome (Figure 1A).

Among them, the expression of *Tle4z1* appears male preference in ESCs and PGCs (Figure 1B), and we speculate that it may play a certain role in sex determination and differentiation. Further quantitative results showed that the expression of *Tle4z1* in males was significantly higher than that in females in the stage of PGCs (*P* < 0.0001) (Figure 1C). Detection of adult chicken in different tissues also found that its specific high expression in testicular tissue, and the expression of *Tle4z1* in male gonads was higher than that of female (*P* < 0.01) (Figure 1D). This phenomenon of sex preference expression also exists in the gonads of chicken embryos. From the development of chicken embryo E4.5, the level of *Tle4z1* mRNA expression in male chicken embryo was significantly higher than that in female (*P* < 0.0001) (Figure 1E). These results suggest that *Tle4z1*, as a male-specific gene, may be involved in the process of male sex differentiation in chicken embryos.

### Construction of Overexpression and Interference Lentiviral Vector of *Tle4z1*

**FIGURE 2:**
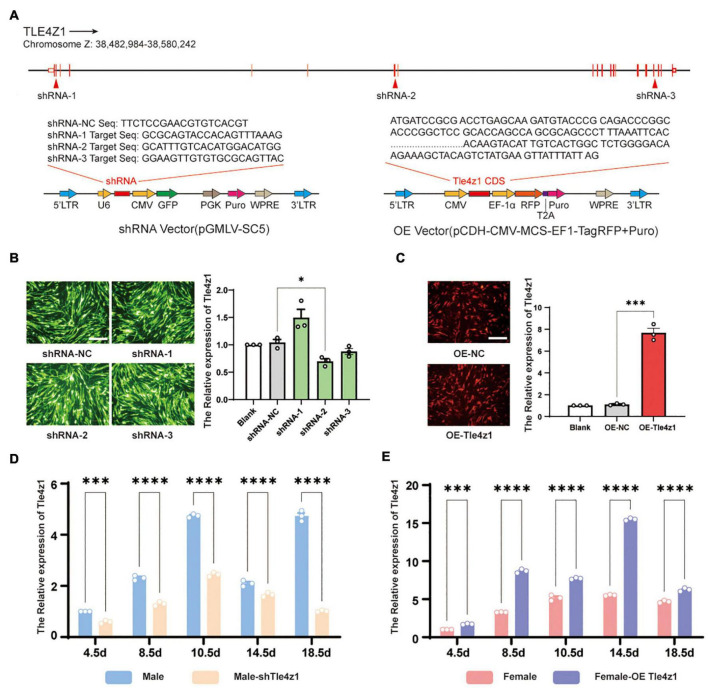
Construction and activity verification of *Tle4z1* RNA interference/overexpression vector. **(A)** RNA interference and overexpression vector design of *Tle4z1*. **(B)** The GFP fluorescence was detected in DF-1 cells *in vitro* indicating effective lentivirus infection. Relative expression of *Tle4z1* in DF1 cells transfected with lentiviral shRNA vectors for 48 h as measured by qRT-PCR. 2#shRNA vector has best effect on interfering the expression of *Tle4z1*. Scale bars = 100 μm. **(C)** The RFP fluorescence was detected in DF-1 cells *in vitro* indicating effective lentivirus infection. Relative expression of *Tle4z1* in DF1 cells transfected with lentiviral overexpression vector for 48 h as measured by qRT-PCR. Scale bars = 100 μm. **(D)** Compared with normal male chicken embryos, the relative expression of *Tle4z1* in male chicken embryos at different stages of development after blunt end injection of lentiviral interference vector *in vivo*. **(E)** Compared with normal female chicken embryos, the relative expression of *Tle4z1* in female chicken embryos at different stages of development after blunt end injection of lentiviral overexpression vector *in vivo*. *P < 0.05, ^**^P < 0.01, ^***^P < 0.001, ^****^P < 0.0001. All values are mean ± SEM. *n* = 3.

In order to verify the function of *Tle4z1* in the process of sex differentiation of chicken embryo, lentivirus interference and overexpression vector of *Tle4z1* were designed and constructed (Figure 2A). The constructed vector with GFP fluorescent protein labeling was transfected into DF-1 cells. A large number of fluorescent expressions was observed 48 h later, indicating that the vector can be effectively expressed *in vitro*. The result showed that after interfering with *Tle4z1*, the level of *Tle4z1* mRNA was downregulated. The interference activity of shRNA2-*Tle4z1* was the strongest among the three shRNA targets (*P* < 0.05), so the target was selected to package lentivirus for follow-up experiments (Figure 2B). Meanwhile, the expression of *Tle4z1* increased significantly after overexpression (*P* < 0.001) (Figure 2C). Further experiments showed that in the E4.5-E18.5 of chicken embryos, the mRNA level of *Tle4z1* in female gonads of overexpression group was significantly higher than that of control group *in vivo* (*P* < 0.001) (Figure 2E), and *Tle4z1* was inhibited after interference treatment in male gonads (*P* < 0.001) (Figure 2D), indicating that the recombinant vector could effectively interfere/overexpress *Tle4z1* during chicken embryo development.

### *Tle4z1* Promotes Virilization of Chicken Embryonic Gonadal Phenotype

**FIGURE 3:**
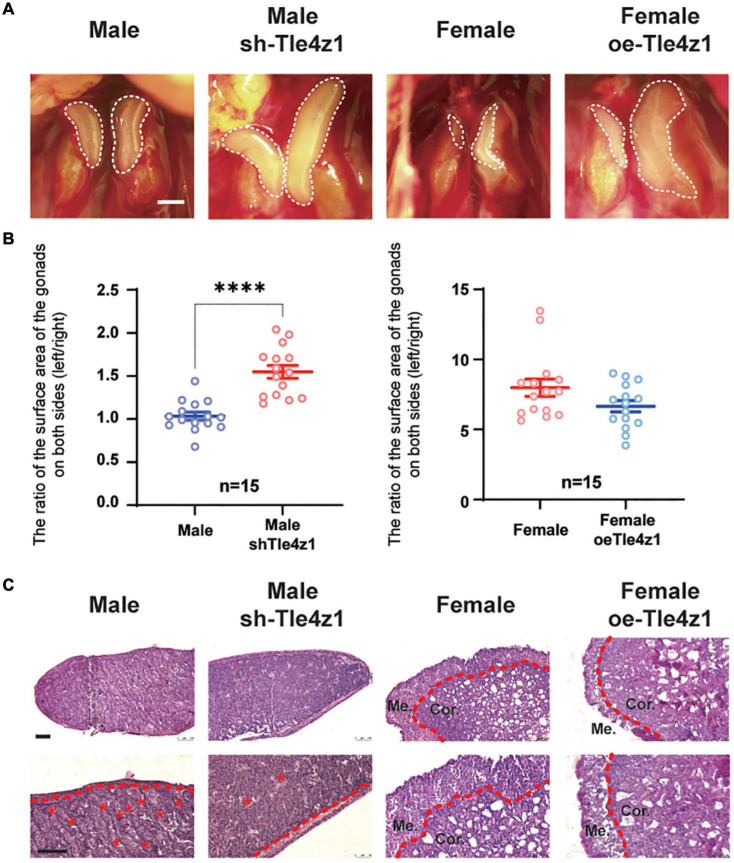
*Tle4z1* promotes the differentiation of chicken embryo gonadal phenotype to male. **(A)** Phenotypic differentiation of gonadal morphology at E18.5. Left two pictures are the comparison of the morphological development difference of chicken embryo testis between control and interference group. Right two are ovarian morphology of the control and overexpression group. Scale bars = 2 mm. **(B)** Statistical analysis of the asymmetric development of the gonads on both sides of chicken embryos. The X-axis represents the group, and the Y-axis represents the ratio of the surface area of the gonads on both sides of the chicken embryo (left/right). ^****^P < 0.0001, values are means ± SEM. *n* = 15. **(C)** Photomicrographs of hematoxylin/eosin (H/E) staining of gonads cultured at E18.5. The dashed red line indicates the border between the medulla and cortex. Cor.: cortex layer; Me.: medulla layer; *tubuli seminiferi contorti; Scale bar = 100 μm.

In order to explore the effect of *Tle4z1* on the phenotypic differentiation of gonads, the gonads of chicken embryos hatched to 18.5 days after blunt injection of vectors were observed (Figure 3A). The statistical results showed that after interfering with the expression of *Tle4z1*, there was a significant asymmetric development of bilateral testes in the experimental group compared with the control group (*P* < 0.0001), while the atrophy of the right ovary in the experimental group was alleviated after overexpression of *Tle4z1* (Figure 3B). HE staining of the gonadal sections showed that the female gonads overexpressing *Tle4z1* were significantly thinner than normal female embryos, showing male characteristics, while the cortex of gonads after interference with *Tle4z1* was significantly thicker, showing female-related characteristics (Figure 3C). These results indicate that *Tle4z1* plays an important role in the development of gonads in chicken embryo.

### *Tle4z1*—a Key Gene That Influences Sex Differentiation to Male

**FIGURE 4:**
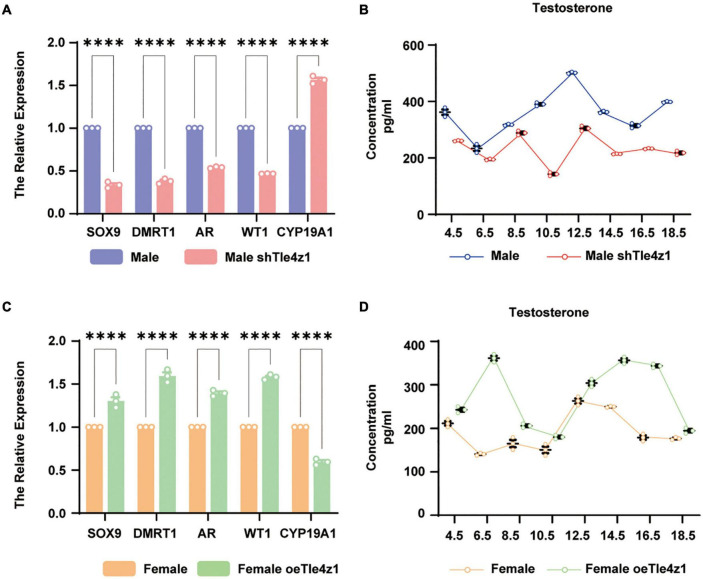
*Tle4z1* promotes the sex differentiation of chicken embryos into males at mRNA and hormone levels. **(A)** Relative expression of sex-related genes (Sox9, DMRT1, AR, WT1 and CYP19A1) in male interference group at E18.5 by qRT-PCR. ^****^P < 0.0001, values are means ± SEM. *n* = 3. **(B)** Changes in the expression of testosterone in male interference group at E4.5, E6.5, E8.5, E10.5, E12.5, E14.5, E16.5, and E18.5 was detected by ELISA. **(C)** Relative expression of sex-related genes in female overexpression group at E18.5 by qRT-PCR. **(D)** Changes in the expression of testosterone in female overexpression group at E4.5, E6.5, E8.5, E10.5, E12.5, E14.5, E16.5, and E18.5 was detected by ELISA.

In this study, it has been found that *Tle4z1* can affect the gonadal phenotypic differentiation of chicken embryos. We detected the sex differentiation genes by qRT-PCR. The results showed that at E18.5, the expression of genes related to male sex differentiation like *Dmrt1*, *AR*, *WT1* and *Sox9* in the gonads of ZZ embryos in treated group of interference decreased significantly, while the mRNA level of *Cyp19a1*, which dominate female differentiation, was significantly higher than that in normal ZZ embryos (*P* < 0.0001) (Figure 4A). After overexpressing *Tle4z1*, the expression of genes related to male differentiation, like *Dmrt1*, *AR*, *WT1* and *Sox9* increased significantly, while *Cyp19a1* decreased significantly (*P* < 0.0001) (Figure 4C). The above results suggest that regulating the expression of *Tle4z1* has an important effect on the sex differentiation of chicken embryos.

Recently, studies have shown that hormones are involved in the process of sex determination and differentiation of chicken embryos (Morris et al., 2018; Wang et al., 2019). For further research on the role of *Tle4z1* in the process of sex differentiation of chicken embryos, this study also detected the changes of sex hormone levels during incubation E4.5-E18.5 after interfering or overexpressing *Tle4z1* by ELISA. The results showed that testosterone in ZZ chicken embryos interfering with *Tle4z1* expression decreased significantly and reached the lowest point at E18.5 (Figure 4B). On the contrary, the level of testosterone in gonad of ZW chicken embryo in overexpression treatment was significantly higher than that in normal female chicken embryo (*P* < 0.01) (Figure 4D). These results suggest that *Tle4z1* can regulate the secretion of testosterone and play an important role in the male differentiation of chicken embryos.

## Discussion

Scientists have never stopped exploring the topic of sex determination and sex differentiation in poultry. In mammals, sex determination and gonadal differentiation have been well-studied, but the unique egg-laying characteristics have hindered research in birds. In recent years, many key genes have also been discovered in sex differentiation. CRISPR/Cas9 system was used to find out that after knocking out DMRT1, the ZZ embryo testis had sex reversal, indicating that DMRT1 is crucial for testis development, and demonstrating the avian sex-determining mechanism is based on DMRT1 dosage (Ioannidis et al., 2021). Similarly, disruption of DMRT1 results in gonad feminization during the early stages of rooster development (Lee et al., 2021). Moreover, DMRT1 can directly or indirectly activate the expression of male factors HEMGN, SOX9 and AMH (Estermann et al., 2021). FOXD1 is an important marker of Sertoli cells upstream of SOX9 expression, and synergistically affects the differentiation and development of chicken embryo testis with Sox9 (Yu et al., 2019). Here, we successfully screened and identified a novel gene, *Tle4z1*, which plays an important role in male differentiation in chicken embryos. Functional validation results showed that interfering with *Tle4z1* could inhibit normal sex differentiation in males, making them appear female, while overexpression of *Tle4z1* could masculinize female embryos. At the same time, we found that *Tle4z1* can regulate the expression of male marker genes such as *Dmrt1, AR, WT1, Sox9* and testosterone.

Existing studies have shown that Tle4 generally affects the development of ear, eye and dorsal ventral nerve cells in the form of corepressor factor, but there are few studies on chickens (Zhu et al., 2002; Bajoghli et al., 2005; Tsuji and Hashimoto, 2005). We detected the expression of *Tle4z1* in eyes, ears and brain of chickens, but it has no significant differential expression between male and female chickens in this study. We found that *Tle4z1* was highly specifically expressed in male and female ESCs and PGCs. In combination with its expression of chicken embryo development stages, *Tle4z1* was significantly more expressed in male than female chicken embryo gonads, suggesting that *Tle4z1* may be involved in the differentiation process of male chicken embryos. Chicken is a typical model animal of asymmetric gonadal development. When chicken embryo develops to E8, the phenotypic changes of ovary can be distinguished, that is, the left ovary develops normally and the right ovary atrophies gradually. Injection of aromatase (*Cyp19a1*) into chicken embryos could reverse the embryonic phenotype of ZZ chicken (Hirst et al., 2018), but it had no effect on the gonadal morphology of ZW type. This phenomenon could only be temporarily affected by cell autonomy. In this study, we found that the asymmetric development of bilateral gonads of E18.5, ZW embryos was significantly improved after overexpression of *Tle4z1*, and the gonadal development of ZZ embryos showed a trend of asymmetric development in the interference group, which indicated that *Tle4z1* could play a role in the gonadal development of both sexes and made the sexual phenotype of chicken embryos develop to male. The key role of this gene in the process of sex differentiation of chicken embryos was further verified by lentivirus interference and overexpression system. It was found that after interfering with the expression of *Tle4z1* gene, the expression of male-specific genes *Dmrt1*, *WT1*, *Sox9* was down-regulated, while the expression of female differentiation gene *Cyp19a1* was up-regulated. The results of this study fully proved that the differentiation of *Tle4z1* gene in the male gonad of chicken embryo is necessary.

In addition, sex differentiation and the maintenance of gonadal phenotype need to be regulated by hormone levels, and sex hormones play an indispensable role in maintaining the development of chicken embryos. The expression level of sex hormones in chicken embryos plays a key role in the sex differentiation of chicken embryos (Wang et al., 2019), so sex determination theory of TDF gene and the theory of cell autonomous sexual development are inseparable from the regulation of hormones, and the development of secondary sexual characteristics needs hormones to maintain. In our research, we detected the effect of *Tle4z1* on testosterone level, and found that with the change of *Tle4z1* gene expression, the testosterone level was always positively correlated with the expression level of *Tle4z1*. Androgen receptor gene *AR* is the target gene of androgen regulation, and testosterone can function through the targeted receptor gene *AR* (Wrobel et al., 2020). The expression of *AR* was detected at the transcriptional level, and it was found that it was also consistent with the change trend of *Tle4z1* gene expression. All above shows that *Tle4z1*, as a key gene of male differentiation, participates in the process of sex differentiation of chicken embryos, which provides a theoretical basis for further exploration of the molecular mechanism of sex differentiation of chickens (Jin et al., 2020b; Jiang et al., 2021; Zhang et al., 2021).

In sum, we successfully screened and verified the key role of *Tle4z1* in the process of sex differentiation from three aspects of morphology, mRNA and hormone, and revealed its function of promoting sex differentiation to male of chicken embryos, which provided new insights into the molecular mechanism of chicken sex differentiation.

## Data Availability Statement

The data presented in the study are deposited in the Sequence Read Archive (SRA) database at National Center for Biotechnology Information (NCBI) repository, accession number PRJNA608148.

## Ethics Statement

The animal study was reviewed and approved by Ethics Committee of Yangzhou University for Laboratory and Experimental Animals.

## Author Contributions

BL, GC, and YZ contributed to conception and design of the study. KJ and QZ organized the database. XG, ZL, and CH performed the statistical analysis. JJ helped to perform the analysis with constructive discussions. CC performed the experiment and wrote the first draft of the article. CC and SZ wrote sections of the article. All authors contributed to article revision, read, and approved the submitted version.

## Conflict of Interest

The authors declare that the research was conducted in the absence of any commercial or financial relationships that could be construed as a potential conflict of interest.

## Publisher’s Note

All claims expressed in this article are solely those of the authors and do not necessarily represent those of their affiliated organizations, or those of the publisher, the editors and the reviewers. Any product that may be evaluated in this article, or claim that may be made by its manufacturer, is not guaranteed or endorsed by the publisher.

## Abbreviations

AR: androgen receptor
Cyp19a1: cytochrome P450 family 19 subfamily A member 1
Dmrt1: doublesex and mab-3 related transcription factor 1
ESCs: Embryonic Stem Cells
E0: the 0 embryonic day
E4.5: the 4.5th embryonic day
E18.5: the 18.5th embryonic day
MOI: multiplicity of infection
PGCs: Primordial Germ Cells
qRT-PCR: Quantitative Real-time PCR
Sox9: SRY-box 9
*Tle4z1*: transducin like enhancer of split 4
WT1: Wilms tumor 1
sh- *Tle4z1*: shRNA- *Tle4z1*
oe- *Tle4z1*: overexpression- *Tle4z1.*
