# Supplementary material for: Tle4z1 Facilitate the Male Sexual Differentiation of Chicken Embryos
Source: Front Physiol. 2022 Apr 7;13:856980. doi: 10.3389/fphys.2022.856980 (PMC9022655; doi:10.3389/fphys.2022.856980)
Supplement: Supplementary file 1 [file Data_Sheet_1.doc]

**Supplemental data**

**Table 1 *Tle4z1* shRNA target sequence.**

| NO. | TargetSeq |
| --- | --- |
| 1 | GCGCAGTACCACAGTTTAAAG |
| 2 | GCATTTGTCACATGGACATGG |
| 3 | GGAAGTTGTGTGCGCAGTTAC |
| NC | TTCTCCGAACGTGTCACGT |

**Table 2 shRNA oligo sequence.**

| Name | OligoSeq 5’to 3’ |
| --- | --- |
| Primer-T1 | GATCCGCGCAGTACCACAGTTTAAAGTTCAAGAGACTTTAAACTGTGGTACTGCGCTTTTTTG |
| Primer-B1 | AATTCAAAAAAGCGCAGTACCACAGTTTAAAGTCTCTTGAACTTTAAACTGTGGTACTGCGCG |
| Primer-T2 | GATCCGCATTTGTCACATGGACATGGTTCAAGAGACCATGTCCATGTGACAAATGCTTTTTTG |
| Primer-B2 | AATTCAAAAAAGCATTTGTCACATGGACATGGTCTCTTGAACCATGTCCATGTGACAAATGCG |
| Primer-T3 | GATCCGGAAGTTGTGTGCGCAGTTACTTCAAGAGAGTAACTGCGCACACAACTTCCTTTTTTG |
| Primer-B3 | AATTCAAAAAAGGAAGTTGTGTGCGCAGTTACTCTCTTGAAGTAACTGCGCACACAACTTCCG |
| Primer-NC-T | gatcTGTTCTCCGAACGTGTCACGTTTCAAGAGAACGTGACACGTTCGGAGAATTTTTTc |
| Primer-NC-B | aattgAAAAAATTCTCCGAACGTGTCACGTTCTCTTGAAACGTGACACGTTCGGAGAACa |

**Table 3 Sequence of quantitative primer.**

| NO. | Name | PrimerSeq |
| --- | --- | --- |
| 1 | Actin | F: CAGCCATCTTTCTTGGGTAT  R: CTGTGATCTCCTTCTGCATCC |
| 2 | *AR* | F: GCCACTATGGAGCCCTCA  R: CCCGCCTCGTAGCACTT |
| 3 | *CYP19A1* | F: TGTTCCATCACGCTATTT  R: GATTCTTGTTTGGGCTTC |
| 4 | *Dmrt1* | F:AGCGGCAGCGGGTGAT  R: TGGGCAGGGGTACAGG |
| 5 | *Sox9* | F:AAGGAAGCTGGCTGACC  R: CAGCGCCTTGAAGATGG |
| 6 | *Tle4z1* | F:GCTCCGCACCAGCCAGC  R:CATGACATAATGCCGCTGCATCTC |
| 7 | *WT1* | F:GGCCATACGACAGGATA  R:CGGACAATAGTCGGAGC |

**
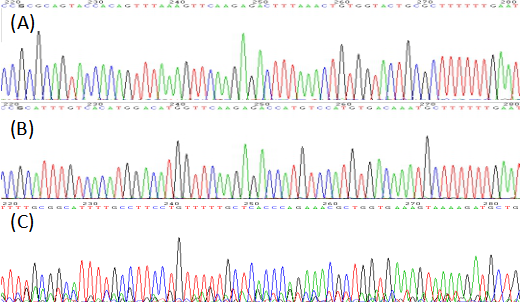
**

**Figure 1 Construction of interference vector.**

The result of shRNA sequencing alignment.A. shRNA1-*Tle4z1*; B. shRNA2-*Tle4z1*; C. shRNA3-*Tle4z1.*

**
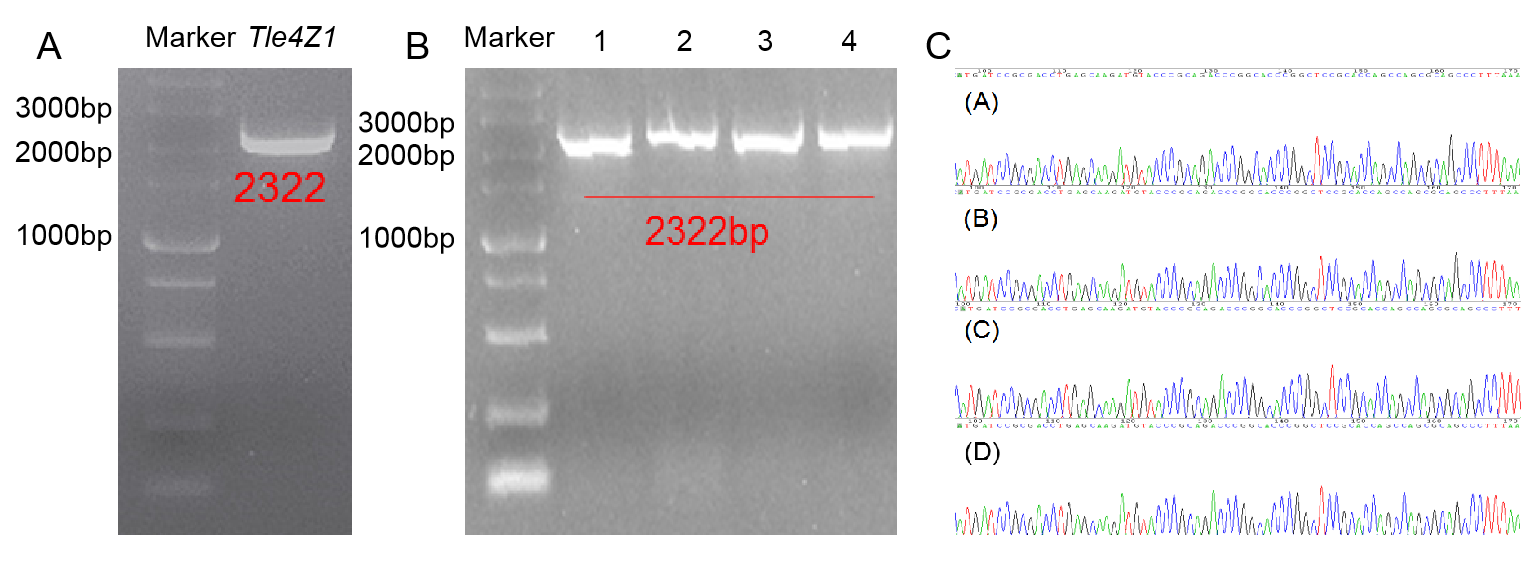
**

**Figure 2 Construction of overexpression vector.**

1. Amplification of the CDS sequence of *Tle4z1;* B. Bacterial fluid PCR Detection. 1:OE1-*Tle4z1*; 2: OE2-*Tle4z1*; 3: OE3-*Tle4z1*; 4: OE4-*Tle4z1*. Marker:5000bp DNA Ladder; C.Sequencing comparison chart of overexpression vector. (A) OE1-*Tle4z1*; (B) OE2-*Tle4z1*; (C) OE3-*Tle4z1*; (D) OE4-*Tle4z1*
